# Optimizing diagnosis of *Mycobacterium tuberculosis* latent infection: ELISpot as a technique of choice for samples with high negative control values in QuantiFERON assay

**DOI:** 10.1128/spectrum.01281-26

**Published:** 2026-10-06

**Authors:** Axelle Amen, Chantal Dumestre-Pérard, Edouard Bonneville, Antoine Vilotitch, Martine Pernollet, Giovanna Clavarino

**Affiliations:** 1 Department of Immunology, Grenoble Alpes University Hospital, University of Grenoble Alpes, CNRS, CEA, UMR5075, IBS 548172, Grenoble, France; 2 Department of Immunology, Grenoble Alpes University Hospital 548172, Grenoble, France; 3 Data Engineering Unit, Public Health Department, Grenoble Alpes University Hospital, University of Grenoble Alpes 36724, Grenoble, France; Indian Institute of Science Education and Research Bhopal, Bhopal, Madhya Pradesh, India

**Keywords:** ELISpot, QuantiFERON, IGRA test, *Mycobacterium tuberculosis*, latent tuberculosis, LTBI

## Abstract

A quarter of the world’s population is considered to be affected by latent tuberculosis infection (LTBI) by the World Health Organization, which recommends that individuals with LTBI receive anti-tuberculosis treatment, aiming to avoid progression to active tuberculosis and transmission. Moreover, patients undergoing immunosuppressive therapy, such as anti-TNFα antibodies, constitute a growing high-risk group for which LTBI screening prior to treatment initiation is strongly advised. Improving LTBI diagnosis and patient management relies on the accuracy of broadly deployed laboratory tests, such as interferon-gamma release assays (IGRAs). The QuantiFERON-TB test is one of the most widely used IGRAs worldwide. In some cases, results may be defined as indeterminate when the negative control (Nil) exceeds 8 IU/mL, delaying or preventing appropriate patient care. This scenario was observed in 57 (0.8%) patients out of the 6,600 tested in Grenoble University Hospital from November 2022 to November 2025. Subsequent re-evaluation with either QuantiFERON-TB and/or the ELISpot-based T-SPOT.TB assays was possible for 34 patients. Among the 15 samples tested again with QuantiFERON-TB, 12 finally resulted in being positive, while three remained indeterminate. By contrast, all 19 patients tested with T-SPOT.TB returned unequivocally positive results. We suggest that repeating IGRA testing is essential when QuantiFERON-TB results are indeterminate due to elevated Nil values, with the T-SPOT.TB assay being a choice solution. Our results highlight an impactful strategy for strengthening LTBI diagnosis, enabling timely chemoprophylaxis, preventing progression to active disease, and contributing to global tuberculosis control.

**IMPORTANCE:**

Interferon-gamma release assays (IGRAs), including QuantiFERON-TB and ELISpot-based T-SPOT.TB, are essential laboratory tools for latent tuberculosis infection (LTBI) diagnosis. However, indeterminate QuantiFERON results with elevated Nil values (>8 IU/mL) are a challenge to timely clinical management. Our findings demonstrate that systematic retesting with IGRAs is essential, with most cases yielding positive results upon repeat testing. T-SPOT.TB appears to be the most reliable alternative for resolving these indeterminate outcomes. This approach enhances LTBI detection, supports prompt chemoprophylaxis, and contributes to preventing progression to active tuberculosis.

## INTRODUCTION

Latent tuberculosis infection (LTBI) can be defined as a state of immune response to *Mycobacterium tuberculosis* (*Mt*) antigens in the absence of clinical evidence of *Mt* infection, with affected individuals being at increased risk of progressing to active tuberculosis and subsequent transmission. It has been estimated that a quarter of the world’s population is affected by latent tuberculosis infection (1), making accurate diagnosis a cornerstone of tuberculosis control strategies (2, 3).

In high-income countries, screening for LTBI is recommended in groups of presumed immunocompetent subjects at increased risk of having LTBI, such as people migrating from endemic countries or identified contact cases (4). It is also part of the baseline evaluation before immunosuppressive therapies or organ transplantation (5). With the global rise in the use of immunosuppressive agents, now concerning millions of individuals, the importance of LTBI screening is more relevant than ever (6). Especially among patients receiving anti-TNF-α therapy for autoimmune diseases, this screening is essential, as they represent a well-established high-risk population (7). Performing interferon-gamma release assays (IGRAs) is also needed in certain clinical contexts, such as atypical presentations of pulmonary disease, suspected extrapulmonary disease, or paucibacillary forms in children where TB diagnosis is difficult.

IGRAs are the only biological tests available for diagnosing LTBI and detect interferon-gamma (IFN-γ) produced by T lymphocytes following stimulation with peptides specific to the *Mt* complex. Unlike the tuberculin skin test, IGRAs use peptides absent from most environmental mycobacteria and from Bacille Calmette–Guérin (BCG) vaccine substrains, thereby reducing the risk of false-positive results related to prior vaccination (8). Two main IGRA techniques are available: the QuantiFERON-TB Gold Plus (QIAGEN) and the T-SPOT.TB ELISpot assay (Oxford Immunotec Ltd.). The QuantiFERON-TB Gold Plus assay is more widely adopted, as it can be readily automated, and it does not require cell separation or expertise in cell culture, unlike T-SPOT.TB ELISpot (9, 10).

Despite the reliability of the QuantiFERON-TB Gold Plus test, indeterminate results (ITRs) can occur, preventing diagnosis, especially for LTBI. Indeterminate results are challenging for clinical decision-making, particularly when assessing the need for preventive therapy, since they themselves can be a source of adverse effects. The most frequent and most explored cause for ITRs is a failure of cell stimulation in contact with the mitogen in the positive control condition (11). A less frequent and less studied but clinically relevant cause of ITRs comes from an elevated IFN-γ concentration, exceeding 8 IU/mL, in the negative control condition called “Nil” tube. Although some studies have proposed modified laboratory protocols to overcome high-Nil indeterminate results, these approaches are labor-intensive and not readily applicable in routine clinical practice (11). In contrast, our study evaluates a pragmatic strategy based on repeating IGRA testing. To our knowledge, this is the largest cohort to investigate this approach and provides real-world evidence for the management of indeterminate QuantiFERON-TB Plus results associated with elevated Nil concentrations in the diagnosis of *Mycobacterium tuberculosis* infection.

## MATERIALS AND METHODS

### Study population

This monocentric, retrospective study was conducted in the Immunology Department of Grenoble Alpes University Hospital. A total of 6,600 QuantiFERON-TB Gold Plus test results obtained between November 2022 and December 2025 were analyzed. Only samples meeting all required pre-analytical quality criteria and compliance with the recommended incubation time were analyzed and therefore included in the study. Collected data included patient demographics (age, sex, and country of origin) and biological results from the QuantiFERON-TB Gold Plus assay, as well as T-SPOT.TB ELISpot, when available.

### Interferon-gamma release assays

The kit Liaison QuantiFERON-TB Gold Plus (ref 311050, QIAGEN) was used following manufacturer specifications. Briefly, whole blood was directly sampled into four distinct QuantiFERON-TB Gold Plus blood collection tubes (QIAGEN): a Nil tube (negative control), a Mitogen tube containing PhytoHemAgglutinin (PHA, positive control), and two TB antigen tubes (TB1 and TB2). Both TB1 and TB2 contain peptide cocktails from ESAT-6 (Early Secreted Antigen Target 6 kDa) and CFP-10 (10 kDa Culture Filtrate Protein) to stimulate CD4^+^ T-helper lymphocyte responses, while TB2 also includes additional peptides designed to elicit CD8^+^ T-cell responses (12). Cells were incubated 16 to 20 h after collection at 37°C, 5% CO₂. Released IFN-γ was quantified using a sandwich chemiluminescence immunoassay. Mouse monoclonal anti-IFN-γ antibodies were coated on magnetic particles as the solid phase, and detection was performed with a second monoclonal anti-IFN-γ antibody, with signal quantified using Liaison XL (DiaSorin). In order to validate the test, the Nil value has to be ≤8 IU/mL, and the Mitogen response ≥0.5 IU/mL in the absence of reactivity to TB antigens; otherwise, as specified by the manufacturer, the test is considered “Indeterminate.” A test is considered positive if either antigen tube shows IFN-γ release ≥0.35 IU/mL and ≥25% above the Nil value.

T-SPOT.TB ELISpot assay (ref TB.300, Oxford Immunotec) was performed according to the manufacturer’s instructions. Briefly, peripheral blood mononuclear cells (PBMCs) were isolated from whole blood using Ficoll density-gradient centrifugation, following the addition of T-Cell Xtend reagent (ref TTK.610, Oxford Immunotec). Within 32 h of sampling, isolated PBMCs were incubated for 16 to 20 h at 37°C, 5% CO₂, under four conditions: unstimulated (negative control), stimulated with ESAT-6-derived peptides (panel A), stimulated with CFP-10-derived peptides (panel B), or stimulated with PHA (positive control). IFN-γ secreted by activated lymphocytes was captured by monoclonal antibodies coated on the plate and subsequently detected using a secondary anti-IFN-γ antibody, according to the sandwich ELISA principle. Each spot corresponded to a single IFN-γ-producing cell. Plates were read using a Bioreader 7000 (Biosys). Results were interpreted after subtracting the spot count in the negative control well from those of panels A and B. Results were considered interpretable if there were ≤10 spots in the negative control well and ≥20 spots in the positive control. The test was considered positive if both panels A and B showed ≥6 spots above the negative control.

## RESULTS

**Fig 1:**
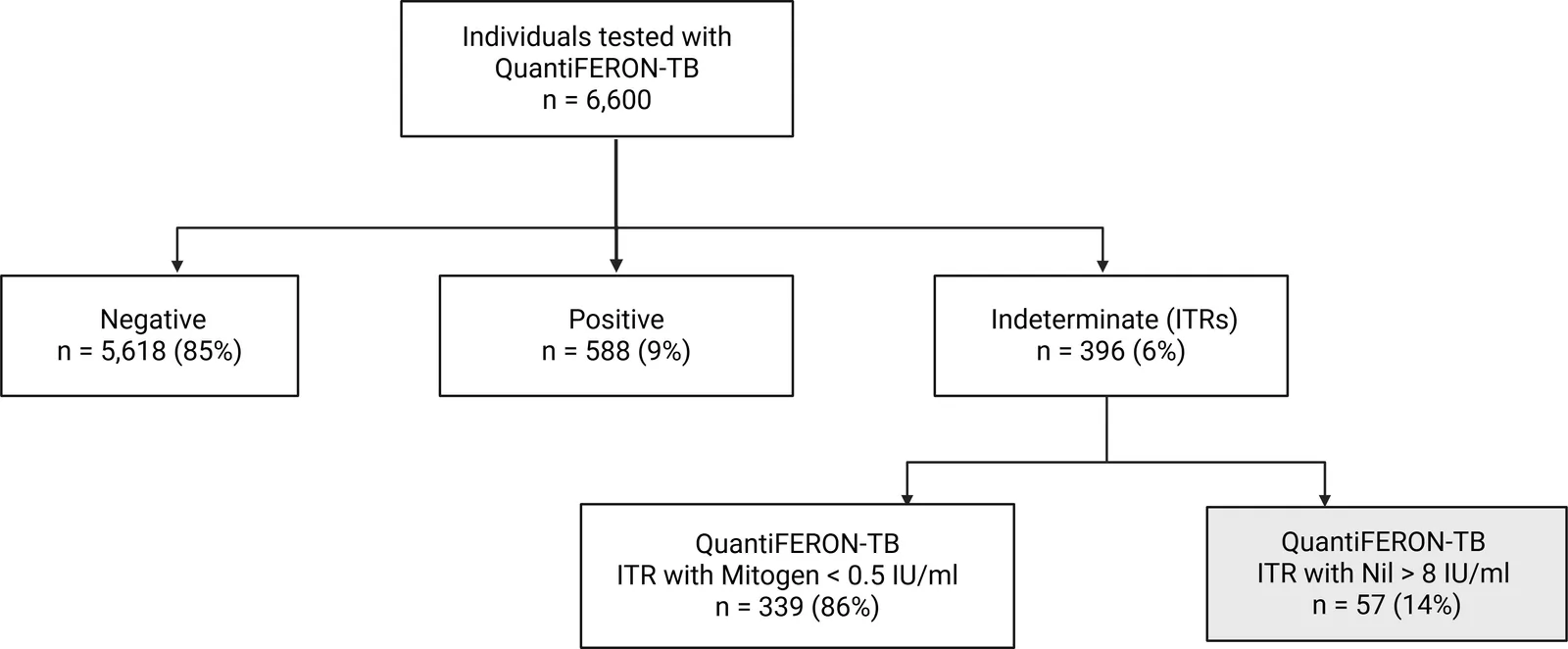
Diagram of QuantiFERON-TB results for the 6,600 subjects tested. Figure was created using Biorender.

A monocentric retrospective descriptive study, including 6,600 subjects tested with QuantiFERON-TB Gold Plus test, was conducted in the Immunology Department of Grenoble Alpes University Hospital, from November 2022 to December 2025. As indicated in Fig. 1, 5,618 (85%) showed negative results, while 588 (9%) showed positive results. Of note, 396 subjects (6%) had indeterminate results. Among the indeterminate cases, 339 (86%) showed no response in the positive control (mitogen) tube, while 57 (14%) had elevated IFN-γ levels in the Nil tube (>8 IU/mL). These latter accounted for 0.8% of total tested samples.

In this precise case, a new blood sampling and repetition of an IGRA test is recommended (13). Among these 57 patients with high IFN-γ levels in the Nil tube, 23 subjects were lost to follow-up, while 34 subjects had a new blood sampling, within a median time lapse of 30 days from the first sampling. According to the choice of prescription of the physician, QuantiFERON-TB Gold Plus was either repeated or replaced by the T-SPOT.TB assay, or both tests were performed. Among the 34 subjects retested, 15 were women and 19 men; the mean age was 43.4 years; 28 subjects (82%) were born in endemic countries for tuberculosis.

**Fig 2:**
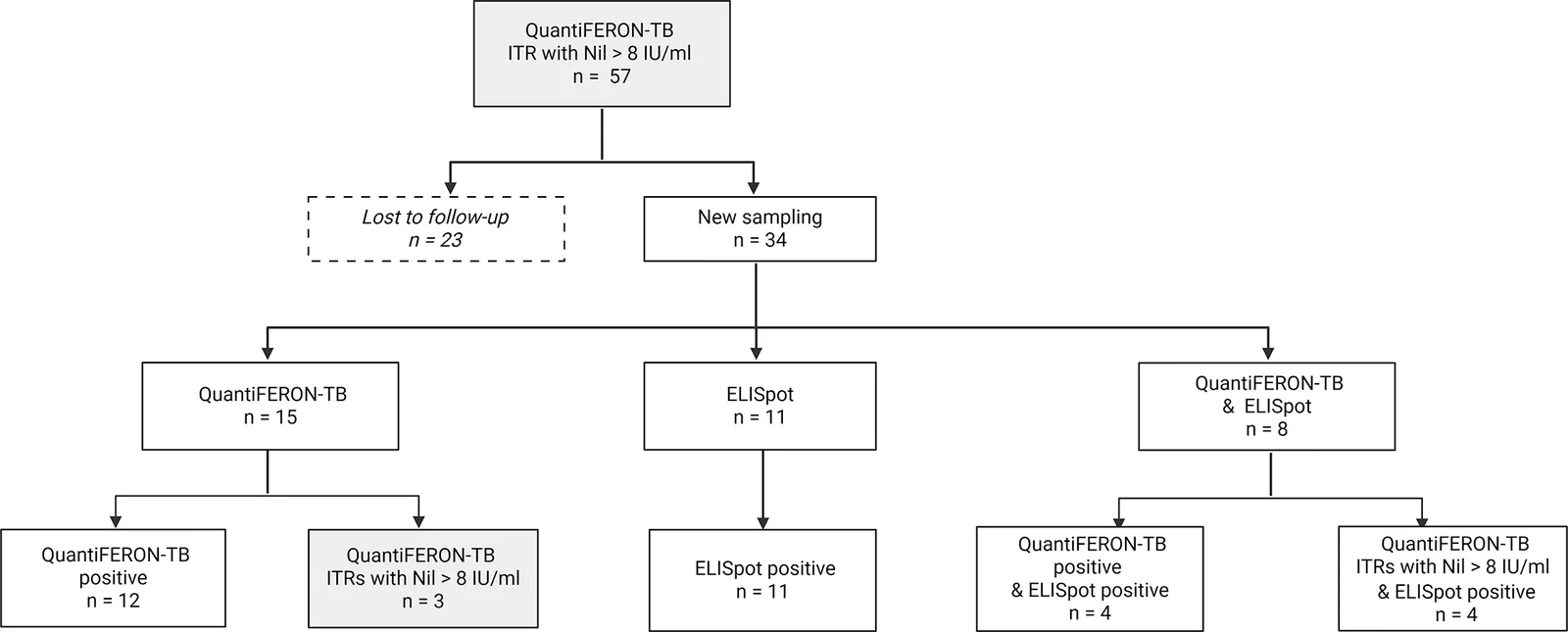
Flow diagram of QuantiFERON and/or ELISpot results for the 57 subjects with ITRs due to Nil >8 IU/mL. Figure was created using Biorender.

As shown in Fig. 2, 15 patients were retested using QuantiFERON-TB alone, 11 using T-SPOT.TB alone, and 8 both with QuantiFERON-TB Gold Plus and T-SPOT.TB. In none of these cases did the repeat testing yield a negative result, regardless of the technique used. Among the 15 subjects retested using QuantiFERON-TB Gold Plus alone, 12 showed a positive result, though still with elevated, but sub-threshold, Nil values (≤8 IU/mL); indeed, the mean Nil value was 3.21 IU/mL (SD = 2.5), notably higher than the average of 0.18 IU/mL observed in the overall cohort (after excluding samples with Nil >8 IU/mL). For three patients, ITR results with high Nil values were still obtained following repetition of the QuantiFERON-TB test. Unfortunately, these patients could not be re-sampled for T-SPOT.TB testing. All of them originated from high-risk epidemic areas, as defined in the WHO report 2025 (14). Among them, one patient had confirmed active lymph node tuberculosis, and the two others were addressed by the French Tuberculosis Control Clinic (CLAT: Centre de Lutte Anti-Tuberculose) with high suspicion of tuberculosis infection. Eleven patients were retested using the T-SPOT.TB assay alone. Interestingly, for all the patients retested using the T-SPOT.TB assay, results returned positive. Finally, eight patients were retested using both QuantiFERON-TB Gold Plus and T-SPOT.TB assay. In four patients, tests were positive and concordant, with an average Nil value of 2.15 IU/mL (SD = 2.8). In the other four, QuantiFERON-TB Gold Plus still yielded indeterminate results, while the ELISpot assay returned positive results, allowing a conclusive diagnosis.

Overall, among all 34 patients with an ITR QuantiFERON-TB Gold Plus result due to a Nil value >8 IU/mL, ELISpot enabled the diagnosis of latent tuberculosis for all the subjects for whom a second blood sampling was possible, an essential finding for patient management and, more broadly, for tuberculosis control.

Additional post hoc analyses were conducted to explore differences in outcomes between the initial test and repeat testing using QuantiFERON-TB Gold Plus on the one hand and T-SPOT.TB on the other. McNemar’s tests for paired data showed a significant difference between the initial result and the repeat test for both methods (*P* < 0.001 in both cases).

To further assess differences between QuantiFERON-TB Gold Plus and T-SPOT.TB for repeat testing, a mixed-effects logistic regression model was considered to account for within-subject correlation. However, model estimation was not feasible due to quasi-complete separation of the data, with no negative results observed for T-SPOT.TB. Therefore, a non-parametric Fisher’s exact test was performed, suggesting a statistically significant difference between the two methods (*P* = 0.011).

## DISCUSSION

Diagnosing LTBI is challenging, as the low bacterial burden presents a major obstacle to direct detection methods, and diagnosis therefore relies on indirect evidence of a cellular immune response to *Mt* antigens. Among the available diagnostic tools, IGRAs have become central to LTBI detection, being more convenient for patients and avoiding interference from BCG vaccination or environmental mycobacteria, unlike the tuberculin skin test. Latent tuberculosis infection detection remains crucial prior to immunosuppressive therapy, particularly before anti-TNF-α treatment, as this cytokine plays a dual role in both the pathogenesis of inflammatory diseases, such as inflammatory bowel disease and rheumatoid arthritis, and in host defense against *Mt* through granuloma formation and maintenance (15).

Of the two commercially available IGRAs, QuantiFERON-TB is by far the most widely used. The preference for QuantiFERON-TB testing can be explained by its lower cost, simplified laboratory workflow, and reduced requirement for specialized expertise in cell culture compared with the ELISpot procedure. This is illustrated, for instance, by the observation that among the laboratories enrolled in the international external quality assessment program UK NEQAS, 366 participants registered the QuantiFERON-TB compared with only 48 (11.6%) for the ELISpot assay. Within the QuantiFERON family, several generations of the assay have been developed since its first introduction in 2001: first, QuantiFERON (QTF), followed by QuantiFERON TB Gold In tubes (QFT-GIT). The latest generation, QuantiFERON-TB Gold Plus (QFT-Plus), approved in 2018, is the one used in our study (16). It differs from earlier versions in the composition of its tubes: one contains long peptides derived from ESAT-6 and CFP-10, designed to induce a specific CD4^+^ T-cell response, while an additional tube includes shorter peptides aimed at stimulating IFN-γ production by both CD4^+^ and CD8^+^ lymphocytes. QFT-Plus has demonstrated higher sensitivity, lower ITRs, and improved reproducibility compared with earlier versions (17–19).

Nevertheless, QuantiFERON results may sometimes be uninterpretable, most commonly due to ITRs. In the vast majority of cases, ITRs arise from insufficient IFN-γ secretion in response to PHA stimulation in the positive control, a situation frequently observed in patients with lymphopenia (20) or immunosuppression (21), such as in HIV^+^ individuals with a low CD4^+^ cell count (22). This phenomenon has also been observed in various physiological contexts, including in young children (23) and the elderly (24).

The less common form of ITRs, marked by elevated Nil values (>8 IU/mL) and which is the focus of this study, is less frequent and less described in the literature. In a study by Peng et al. (11), ITRs were observed in 3.8% of individuals tested with QuantiFERON-TB Gold Plus, with elevated Nil values (>8 IU/mL) accounting for 0.4% of total ITRs; this rate of pooled ITRs is consistent with findings reported by Zhou et al. (25), who identified a comparable frequency of 3.9%. In our study, the overall proportion of ITRs observed was slightly higher, at 6%, while the samples with elevated Nil values accounted for 0.8% of all samples.

Several hypotheses have been proposed to explain the detection of elevated IFN-γ concentrations in the absence of prior antigenic stimulation, although these mechanisms remain speculative and were not investigated in the present study. First, the presence in the sample of heterophilic antibodies, known as endogenous antibodies that can bind non-specifically to animal-derived immunoglobulins used in immunoassays, such as the capture and detection mouse monoclonal antibodies employed in QuantiFERON-TB Gold Plus, may contribute to this phenomenon (26, 27). Second, an increased spontaneous IFN-γ secretion, notably observed in tuberculosis patients, has been described in the study of Jung et al. (28), where a link was found between spontaneous IFN-γ release and the stage of tuberculosis infection—highest in patients with active TB, followed by those with latent TB, and lowest in uninfected individuals, an observation also reported by Hussain et al. (29). Interestingly, in our study, 82% of the retested subjects with high Nil values were born in endemic countries for tuberculosis. Indeed, type II IFN, IFN-γ, is the main cytokine involved in mycobacteria control in humans, since it plays a key role in the activation of phagocytic cells, in particular tissue-resident macrophages, and tuberculosis-infected patients produce high levels of IFN-γ. Interestingly, subjects with hereditary immunodeficiencies involving the IL-12/IFN-γ axis, or with autoantibodies anti-IFN-γ, show an important susceptibility to mycobacterial infections (30).

Similarly, a case report by Hangai et al. underlines a rare occurrence of a patient suffering from miliary tuberculosis with two consecutive QuantiFERON-TB with ITR results due to high IFN-γ Nil concentration (31). Consistent with this observation, suggesting that an activated inflammatory state may contribute to elevated background IFN-γ levels, all three patients in our study with recurrent ITRs had confirmed or highly suspected tuberculosis infection. Several strategies have been proposed to overcome limitations associated with ITRs. Certain adjustments to assay procedures have been shown to reduce circulating IFN-γ levels and subsequently decrease the occurrence of ITRs, such as immediate incubation of samples, with a minimized delay between blood collection and processing (32, 33). In the study by Peng et al. (11), the authors proposed replacing plasma with cell culture medium to reduce circulating IFN-γ levels and thereby lower the rate of indeterminate results, an efficient but labor-intensive solution.

In general, a lack of reproducibility of results from QuantiFERON assays has been pointed out, supporting the necessity to retest the samples in certain cases, in order to enhance LTBI diagnostic accuracy and inform treatment decisions (34, 35). In the specific case of high Nil values, a new blood sampling and repetition of the IGRA test is recommended (13). In this present study, resampling was the chosen strategy for decreasing ITR number. As shown by Bi et al. (36), the repetition of the QuantiFERON test after a delay can resolve indeterminacy in a majority of cases, particularly when performed within a month of the initial specimen.

**Fig 3:**
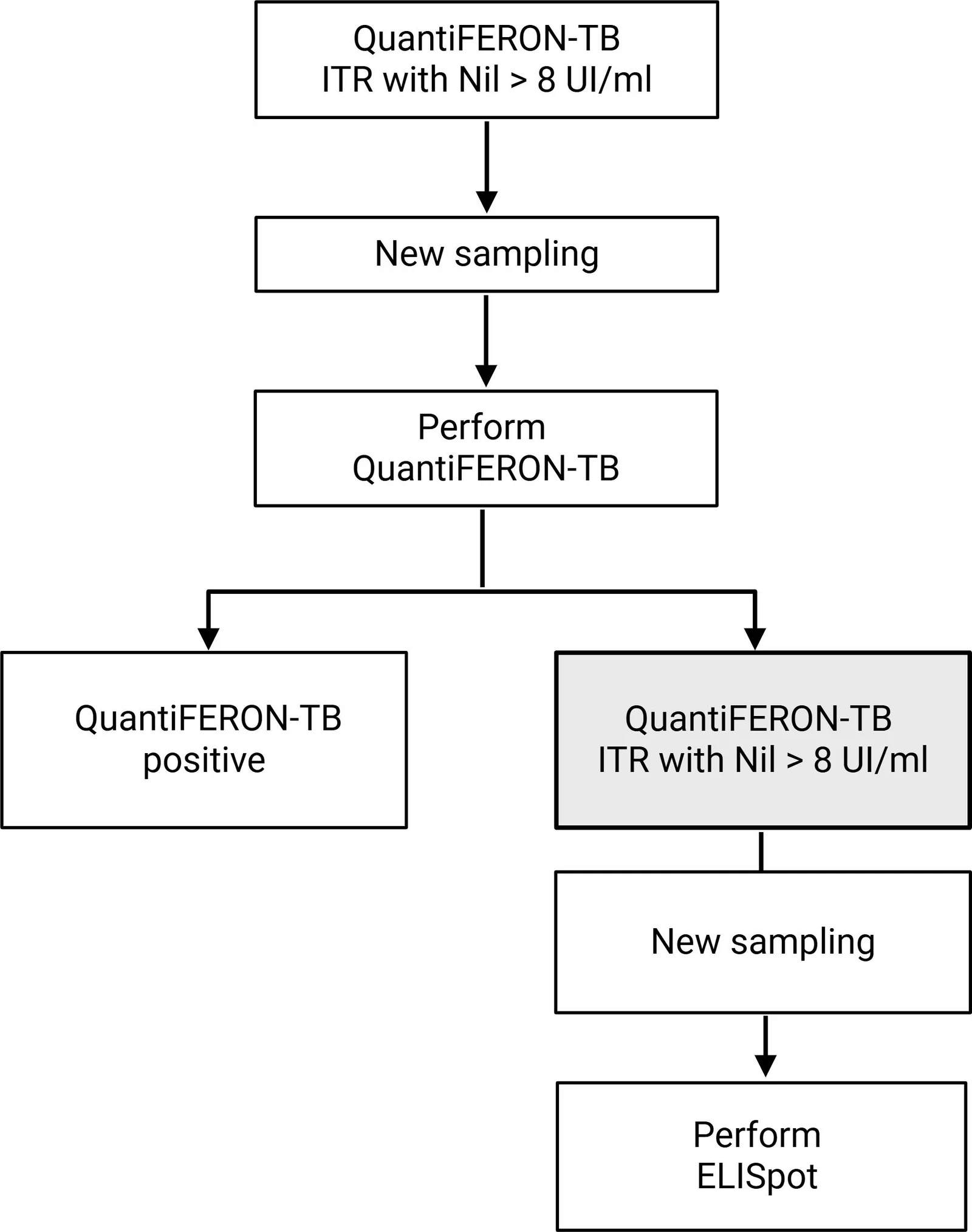
Proposed diagnostic and management strategy for samples with elevated Nil value for the diagnosis of latent *Mycobacterium tuberculosis* infection. Figure was created using Biorender.

Our results suggest that when a QuantiFERON-TB result is indeterminate due to a high Nil value, new sampling is essential, as a positive result is frequently observed upon retesting. Where available, the ELISpot-based T-SPOT.TB assay has to be preferred over the QuantiFERON-TB test. Indeed, in our cohort, repetition of an IGRA test by ELISpot confirmed latent tuberculosis infection in all cases when resampling was possible. This is likely due to the elimination of circulating IFN-γ and heterophilic antibodies during PBMC isolation, thereby minimizing background interference. Additionally, the T-SPOT.TB test is unaffected by immunosuppressive therapy or lymphopenia, both of which are known risk factors for indeterminate results in the QuantiFERON-TB test. Taken together, our results guide experience-based recommendations on the management of ITRs due to high Nil concentration for the diagnosis of *Mt* infection or LTBI, as summarized in Fig. 3. Given the post hoc and exploratory nature of these analyses, these findings should be interpreted with caution and require confirmation in studies specifically designed to address this question.

Interestingly, regarding patients retested only by ELISpot, 4 out of 11 had a final diagnosis of LTBI, and 1 had a diagnosis of active tuberculosis infection; therefore, they were treated accordingly (Table S1). Since clinical and biological information were incomplete for some patients, it would be essential for a next prospective study to collect these data in order to evaluate clinical correlation with results of IGRA techniques and impact on treatment and follow-up of the patients.

Indeed, current IFN-γ-based assays have intrinsic limitations. Notably, some individuals exposed to contagious TB fail to mount detectable IFN-γ responses, yet exhibit T-cell activation markers, such as CD40L (CD154), and corresponding IgM/IgG responses against ESAT-6 and CFP-10, indicating that conventional IGRA assays may miss a subset of infected individuals (37). These observations highlight the need for next-generation diagnostic tools that capture broader aspects of the immune response beyond IFN-γ production (16).

### Conclusion

Pending the development of improved diagnostic approaches, clinicians should be aware that when a QuantiFERON-TB result is indeterminate due to a high Nil value, new sampling is essential, as a positive result is frequently observed upon retesting. Where available, the ELISpot-based T-SPOT.TB assay may represent a valuable alternative, as it yielded interpretable results and supported the diagnosis of LTBI in all resampled patients in our cohort.

Our findings also provide additional evidence supporting the recommendations of the French National Authority for Health (HAS), as published in the Official Journal of the French Republic (13), which state, “In immunocompromised patients (e.g., those with HIV infection or receiving anti-TNF therapy), a negative or indeterminate IGRA result may warrant repeat testing with an IGRA,” as a positive result may prompt chemoprophylaxis to prevent progression to tuberculosis disease.

Overall, our findings suggest an impactful strategy for strengthening LTBI diagnosis, enabling timely chemoprophylaxis, preventing progression to active disease, and contributing to tuberculosis control.

## Data Availability

All data supporting the findings of this study are available within the paper and its Supplemental data file (Table S1).

## SUPPLEMENTAL MATERIAL

Table S1 spectrum.01281-26-s0001.pdf Data of subjects retested only with QuantiFERON (A), only with ELISpot (B), and both with QuantiFERON and ELISpot (C): IGRA results, immunosuppressive status, diagnosis, and TB treatment.

ASM does not own the copyrights to Supplemental Material that may be linked to, or accessed through, an article. The authors have granted ASM a non-exclusive, world-wide license to publish the Supplemental Material files. Please contact the corresponding author directly for reuse.
