# Supplementary material for: Optimizing diagnosis of Mycobacterium tuberculosis latent infection: ELISpot as a technique of choice for samples with high negative control values in QuantiFERON assay
Source: Microbiol Spectr. 2026 Oct 6;14(11):e01281-26. doi: 10.1128/spectrum.01281-26 (PMC13729180; doi:10.1128/spectrum.01281-26)
Supplement: Supplementary file 1 [file spectrum.01281-26-s0001.pdf]

A

QuantiFERON-TB (n =15)					
Initial QFNIL value (UI/ml)	Repeated QFNIL value (UI/ml)	Conclusion	Immunosuppression	Diagnosis	TB treatment
>10	2.13	Positive	no	/	no
>10	4.97		no	/	no
9.37	1.17		no	/	no
>10	2.01		no	/	no
>10	6.58		no		no
>10	2.79		no	/	no
>10	0.26		no	ancient TB treated	in the past
8.59	5.93		no	no	no
>10	7.65		no	no	no
>10	0,25		ND		
>10	1.71		corticosteroids	LTBI	yes
>10	3.10		corticosteroids	/	no
>10	10	ITRs	no	active TB	yes
8.02	8.81		no	/	no
8.41	8.01		ND		

B

ELISPOT (n=11)								
Initial QFNIL value (UI/ml)	Negative control	Panel A	Panel B	Mitogen	Conclusion	Immunosuppression	Diagnosis	TB treatment
>10	1	79	26	341	Positive	no	/	no
>10	0	560	227	673		no	/	yes (before anti-TNFalpha treatment)
8.81	0	143	192	552		no	LTBI	yes
>10	2	314	112	420		ND		
>10	0	4	51	328		ND		
9.79	0	131	340	614		ND		
>10	0	266	218	169		no	active TB	yes
>10	0	201	87	243		no	/	no
>10	1	67	32	158		no	LTBI	yes
>10	0	25	107	220		no	LTBI	yes
>10	0	378	306	243		no	LTBI	yes

C

QuantiFERON-TB & ELISPOT (n=8)										
Initial QFNIL value (UI/ml)	Repeated QFNIL value (UI/ml)	ELISPOT Negative control	ELISPOT Panel A	ELISPOT Panel B	ELISPOT Mitogen	Quantiferon conclusion	ELISPOT conclusion	Immunosuppression	Diagnosis	TB treatment
>10	6.09	0	216	272	134	Positive	Positive	no	active TB	yes
>10	0.03	0	24	22	503			no	/	no
8.33	2.25	0	188	187	459			belatacept (renal transplantation)	/	no
>10	0.26	0	58	5	255			no	/	no
>10	10	1	228	214	333	ITRs		no	/	no
>10	9.25	0	267	327	193			no	/	no
>10	>10	0	249	163	146			no	/	no
>10	>10	0	70	107	341			HIV+	/	no
